# Identification of Novel Deregulated RNA Metabolism-Related Genes in Non-Small Cell Lung Cancer

**DOI:** 10.1371/journal.pone.0042086

**Published:** 2012-08-02

**Authors:** Iñaki Valles, Maria J. Pajares, Victor Segura, Elisabet Guruceaga, Javier Gomez-Roman, David Blanco, Akiko Tamura, Luis M. Montuenga, Ruben Pio

**Affiliations:** 1 Division of Oncology, Center for Applied Medical Research, Pamplona, Spain; 2 Department of Histology and Pathology, School of Medicine, University of Navarra, Pamplona, Spain; 3 Genomics & Bioinformatics Unit, Center for Applied Medical Research, Pamplona, Spain; 4 Department of Pathology, Marques de Valdecilla University Hospital, School of Medicine, University of Cantabria, Santander, Spain; 5 Department of Thoracic Surgery, Clinica Universidad de Navarra, Pamplona, Spain; 6 Department of Biochemistry, School of Sciences, University of Navarra, Pamplona, Spain; Lehigh University, United States of America

## Abstract

Lung cancer is a leading cause of cancer death worldwide. Several alterations in RNA metabolism have been found in lung cancer cells; this suggests that RNA metabolism-related molecules are involved in the development of this pathology. In this study, we searched for RNA metabolism-related genes that exhibit different expression levels between normal and tumor lung tissues. We identified eight genes differentially expressed in lung adenocarcinoma microarray datasets. Of these, seven were up-regulated whereas one was down-regulated. Interestingly, most of these genes had not previously been associated with lung cancer. These genes play diverse roles in mRNA metabolism: three are associated with the spliceosome (ASCL3L1, SNRPB and SNRPE), whereas others participate in RNA-related processes such as translation (MARS and MRPL3), mRNA stability (PCBPC1), mRNA transport (RAE), or mRNA editing (ADAR2, also known as ADARB1). Moreover, we found a high incidence of loss of heterozygosity at chromosome 21q22.3, where the ADAR2 locus is located, in NSCLC cell lines and primary tissues, suggesting that the downregulation of ADAR2 in lung cancer is associated with specific genetic losses. Finally, in a series of adenocarcinoma patients, the expression of five of the deregulated genes (ADAR2, MARS, RAE, SNRPB and SNRPE) correlated with prognosis. Taken together, these results support the hypothesis that changes in RNA metabolism are involved in the pathogenesis of lung cancer, and identify new potential targets for the treatment of this disease.

## Introduction

Lung cancer is one of the most common human cancers and a leading cause of cancer death worldwide [1], [2]. It includes two principal histological subtypes, small cell lung cancer (SCLC) and non-small cell lung cancer (NSCLC), and the latter accounts for 80–85% of all cases. Lung cancer is often detected at an advanced stage, at which point the disease is nearly incurable. Further characterization of the biological alterations associated with its pathogenesis could potentially help identify new biomarkers for early diagnosis and new targets for more effective therapies.

Alternative splicing is a biological process essential for protein diversity. Through alternative splicing, multiple transcripts are generated from a single mRNA precursor. Alterations in alternative splicing have been demonstrated to be associated with various diseases, including cancer. Several alternatively spliced gene products have been linked to the development of neoplastic disease [3]. Cancer-associated splice variants may potentially serve as diagnostic and prognostic tools as well as therapeutic targets in cancer [4]. In lung cancer, many splicing alterations have been previously described in cancer-related processes such as cell growth, cell cycle control, apoptosis, or angiogenesis [5]–[9]. For example, high levels of the anti-apoptotic variant Bcl-xL have been reported to contribute to tumor progression in both SCLC and NSCLC [10], [11]. Recently, splicing changes that affected transcripts of VEGFA, MACF1, APP, and NUMB were demonstrated in patients with lung adenocarcinoma [9]. Moreover, the expression of a specific isoform of NUMB in tumor samples was shown to promote cell proliferation [9]. In addition, modulation of caspase 9 alternative splicing was demonstrated to affect the sensitivity of NSCLC cells to some chemotherapeutic agents [12].

The mechanisms underlying aberrant alternative splicing in lung cancer remain poorly understood. In some cases, mutations in splicing regulatory elements within the nucleotide sequence of the gene result in modifications in splice site selection, in turn leading to alternatively spliced transcripts [13]. In other cases, changes in proteins related to mRNA-metabolism are responsible for the abnormal splice patterns. Some studies have reported changes in the concentration, localization, composition or activity of several RNA-binding proteins in lung cancer [14]–[19], suggesting that this pathway is frequently altered and is important for malignant transformation. The RNA binding protein SF2/ASF is overexpressed in NSCLC tumors and promotes survival by enhancing survivin expression [18]. Studies in animal models suggest that altering the balance between different RNA-binding proteins contributes to lung carcinogenesis [20], [21]. However, characterization of the repertoire of RNA metabolism-related molecules involved in lung cancer is currently incomplete, and further studies are warranted.

In this study, we aimed to identify new RNA metabolism-related genes with altered expression in primary lung tumors. Using lung adenocarcinoma microarray datasets, we searched for genes that exhibited significantly different expression levels between normal and tumor lung tissues. We identified seven mRNA metabolism-related proteins up-regulated in tumor tissues and one down-regulated. Some of the over-expressed genes belong to the family of spliceosomal proteins, implicating this cellular machinery in the development of NSCLC. The downregulated gene ADAR2 was located in an area with a high frequency of deletions in NSCLC. In addition, the expression of five of these genes was associated with the prognosis of patients with lung adenocarcinoma, supporting the importance of RNA metabolism in lung cancer biology.

## Results

### Gene Selection by Bioinformatics Analysis

**Figure 1:**
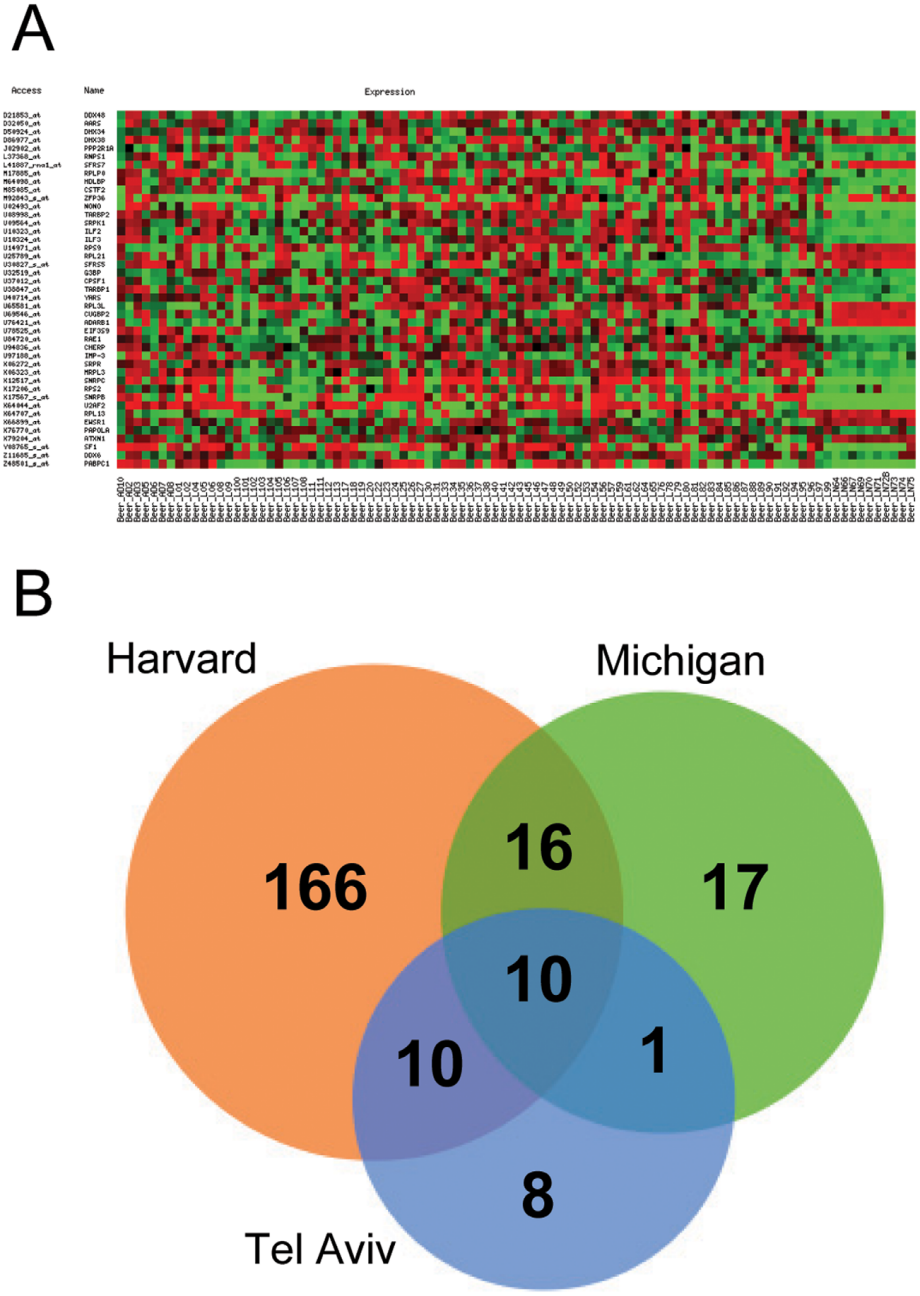
Gene selection by bioinformatics analysis. A ) RNA-related genes with expression levels significantly different (p<0.01) between lung adenocarcinoma samples and normal lung samples (last 10 columns) in one of the microarray databases used in the study [23]. Red color denotes higher expression levels, whereas green color indicates lower expression levels. **B**) Venn diagram corresponding to genes with significant expression differences between normal and adenocarcinoma samples.

Genes belonging to RNA metabolism-related categories were selected from the Gene Ontology database (www.godatabase.org). Three major RNA metabolism-related categories were chosen: “RNA binding”, “RNA splicing”, and “spliceosome complex”. The fold-change (FC) of gene expression levels between normal and adenocarcinoma lung tissue was calculated using data from three microarray experiments [22]–[24]. A set of genes with mRNA expression levels significantly different between normal and cancer tissues was obtained from each of the three microarray experiments (Table S1). A representative example is shown in Figure 1A, and the overlap between the different lists is shown in Figure 1B. Additionally, we found that these genes were differentially expressed in squamous cell carcinoma, small-cell lung carcinoma, and carcinoid cases present in the datasets (data not shown). To corroborate the validity of this selection, additional *in silico* validation was performed with a fourth independent cohort of lung adenocarcinoma patients [25], and the results confirmed the findings of the initial experiment (Table S2).

### Expression of Selected RNA Metabolism-related Genes in Lung Cancer Cell Lines and Normal Lung Primary Cultures

**Figure 2:**
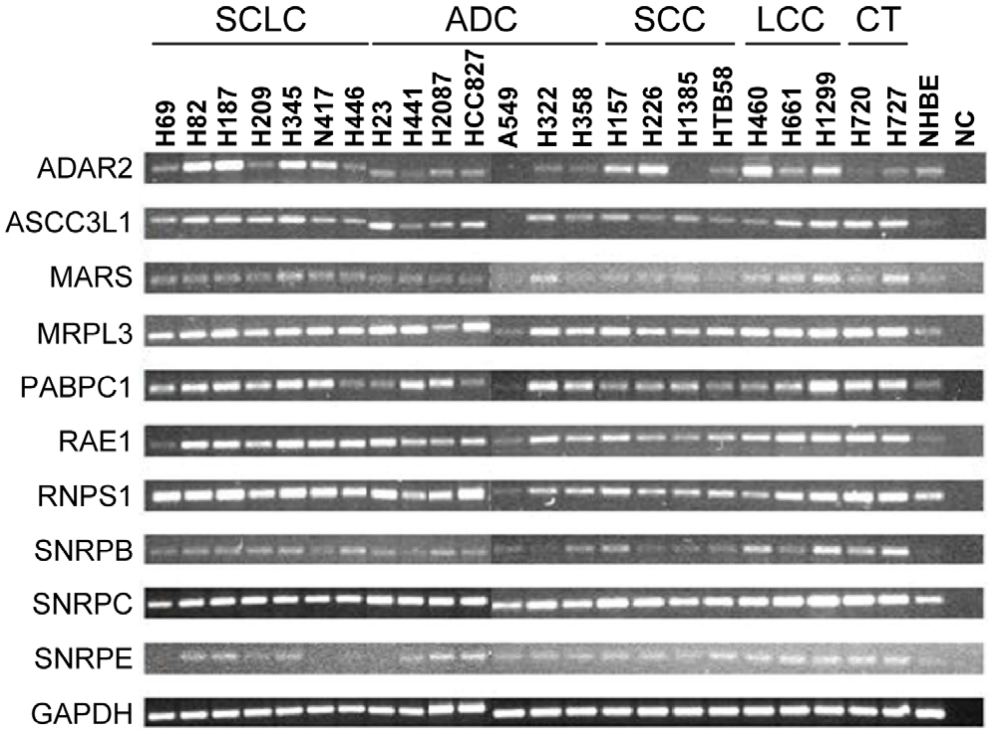
PCR expression analysis of RNA metabolism-related genes. Expression levels were determined in lung cancer cell lines and normal human bronchial epithelial (NHBE) cells. GAPDH was used as control gene. SCLC: small cell lung cancer; ADC: adenocarcinoma; SCC: squamous cell carcinoma; LCC: large cell carcinoma; CT: carcinoid tumor; NC: negative control (water).

Expression levels of the selected genes were evaluated by conventional PCR in SCLC and NSCLC cell lines and in primary NHBE cells. For most genes, mRNA levels in lung cancer cell lines were higher than in NHBE cells (Figure 2). Moreover, mRNA levels were generally higher in SCLC cell lines compared to NSCLC cell lines. This result is in concordance with previous observations in which the expression levels of other RNA-binding proteins were also determined to be higher in SCLC compared to NSCLC [15]. In ADAR2, several lung cancer cell lines exhibited lower mRNA levels compared to NHBE cells. We excluded RNPS1 and SNRPC in subsequent analyses because expression levels were homogeneous among all cell types.

**Figure 3:**
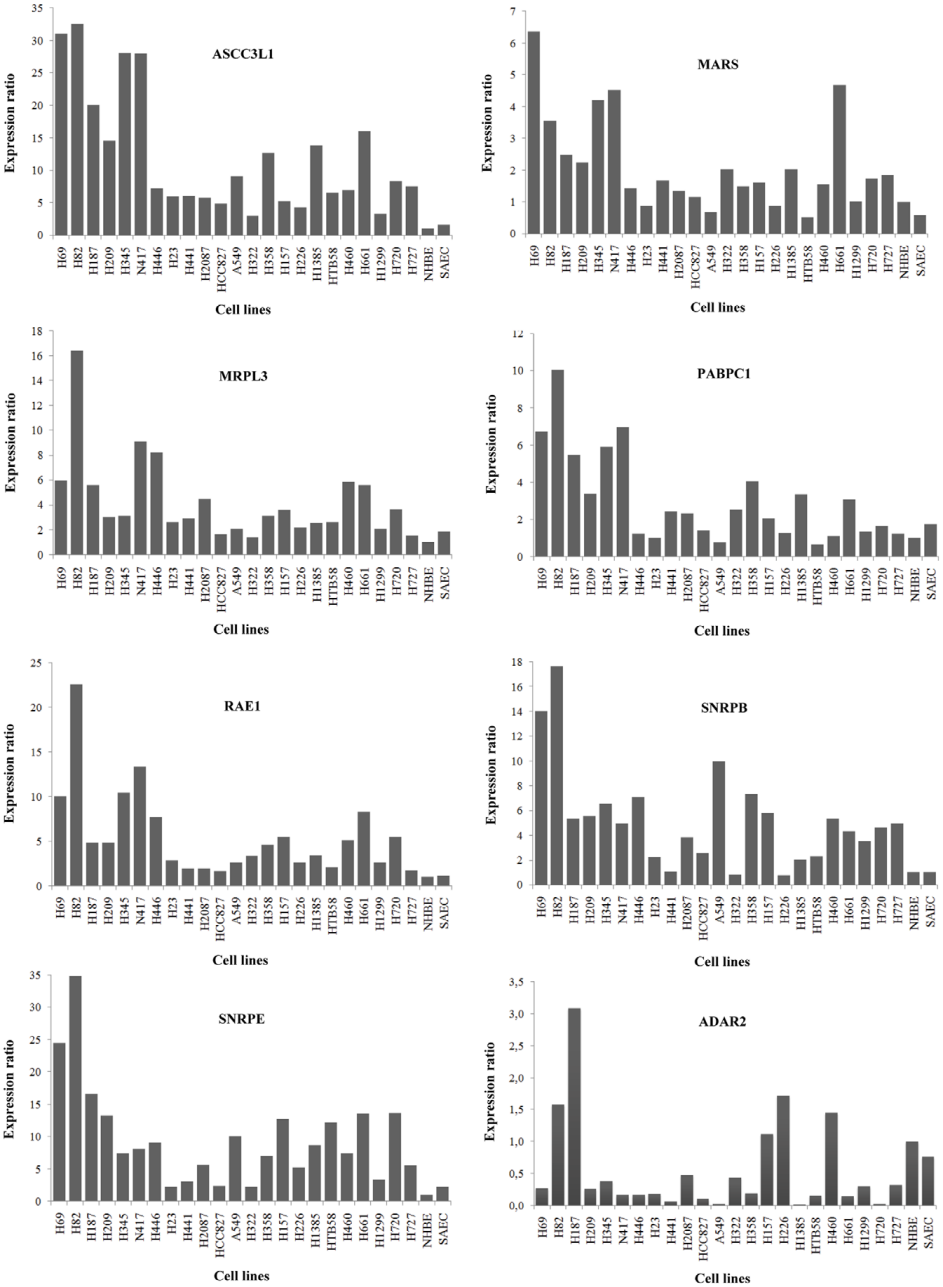
Real time PCR expression analysis of RNA metabolism-related genes. Expression levels were determined in lung cancer cell lines and non-malignant lung primary cultures (NHBE and SAEC). HPRT was used as control gene. Bars represent normalized expression ratios relative to gene levels in NHBE cells. Ratios >1 indicate higher expression levels than in NHBE cells, whereas ratios <1 denote lower expression levels.

To quantify differences in expression levels, real time PCR for each of the eight selected genes was performed in the panel of lung cancer cell lines, as well as in NHBE cells and normal primary SAECs. This analysis confirmed our previous results: expression of the up-regulated genes was higher in lung cancer cells compared to NHBE cells or SAECs. In contrast, ADAR2 expression in lung cancer cells was lower than in NHBE cells or SAECs (Figure 3).

### Genetic Alterations at the ADAR2 Locus

**Figure 4:**
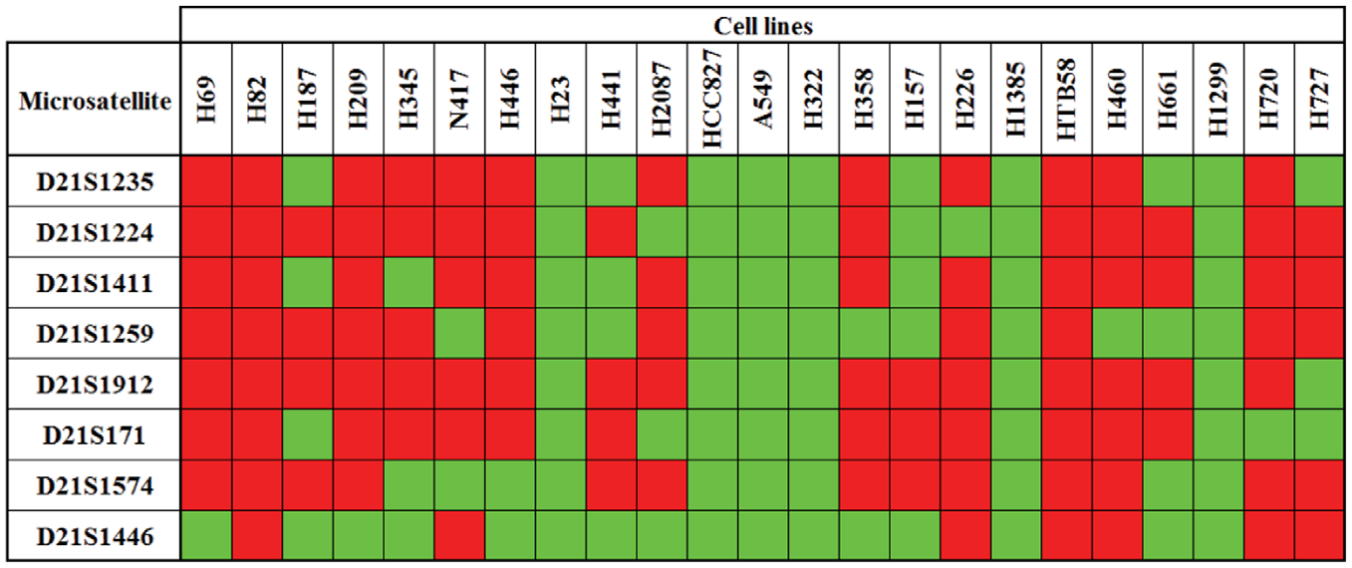
Analysis of microsatellites at 21q22.3 in lung cancer cell lines. Homozygosity (green) or heterozygosity (red) was determined in each cell line. Microsatellites are ordered from the most centromeric to the most telomeric. ADAR2 locus is located within D21S171 and D21S1574.

We characterized the genetic alterations associated with ADAR2 down-regulation in lung cancer. Thus, we evaluated the presence of LOH at 21q22.3, the location of the ADAR2 locus. We used a panel of eight heterozygous microsatellites. Microsatellites were amplified by PCR and analyzed using capillary electrophoresis. Microsatellite heterozygosity was initially evaluated in the panel of lung cancer cell lines and the results are shown in Figure 4. In six cell lines (H23, HCC827, A549, H322, H1385, and H1299), all microsatellites were homozygous. Although the presence of loss of heterozygosity (LOH) is not definitively conclusive due to the lack of matched normal genomic DNA, this homozygosity is extremely unlikely and strongly suggests a loss of genetic material. In other cell lines the results were variable, and certain cases suggested localized areas of LOH (e.g., the most centromeric region in H157 cells). Next, we compared the homozygosity at 21q22.3 and ADAR2 mRNA expression levels (previously obtained). All cell lines homozygous for the microsatellites expressed low ADAR2 mRNA levels. In fact, two of the three cell lines with the lowest ADAR2 mRNA expression levels (A549 and H1385) belong to this group. In contrast, the five cell lines with the highest ADAR2 expression levels (H82, H187, H157, H226 and H460) were mostly heterozygous for the microsatellites flanking the ADAR2 locus (D21S171 and D21S1574).

**Figure 5:**
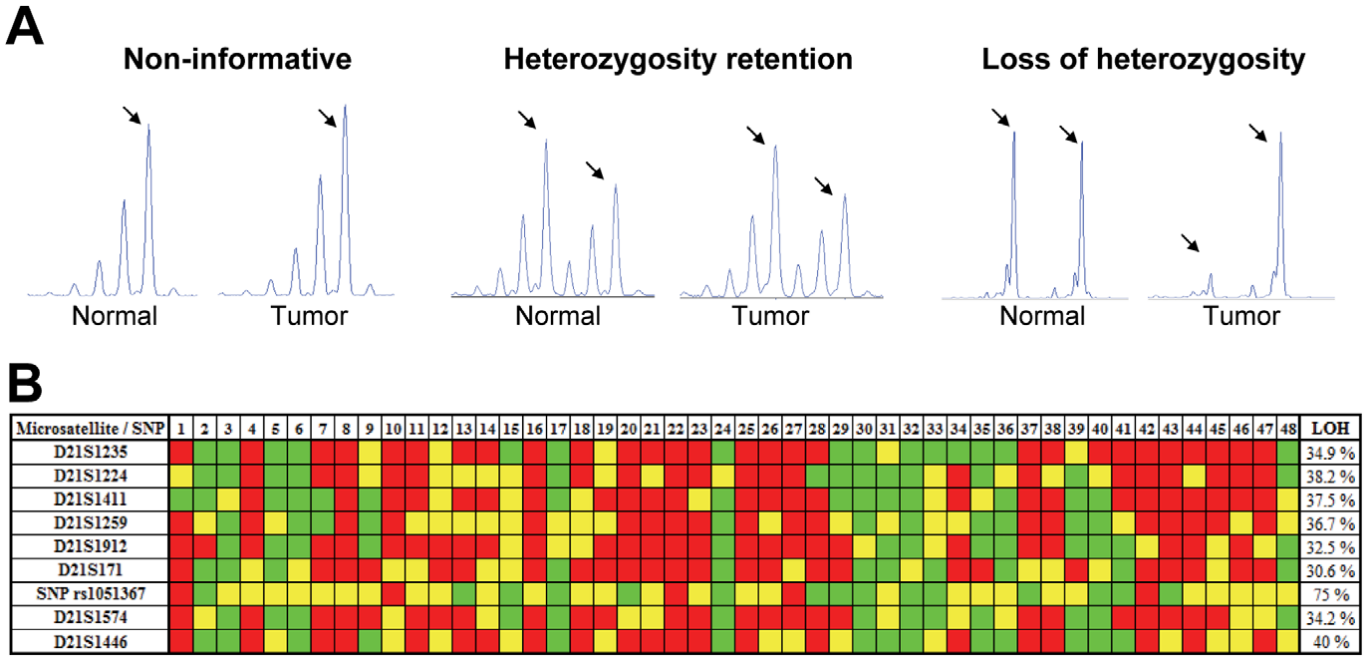
Analysis of LOH at 21q22.3 in lung cancer patients. Genomic DNA from primary lung cancers and their corresponding normal lung tissues were used. **A**) Representative examples of the electrophoretic patterns obtained by microsatellite analysis: a non-informative case, with only one amplification peak; heterozygosity retention, with two peaks in both normal and tumor samples; and LOH, with two peaks in the normal sample but only one peak in the corresponding tumor sample. Arrows point to microsatellite alleles. **B**) LOH of the indicated microsatellites was analyzed in 48 NSCLC patients. Microsatellites are ordered from the most centromeric to the most telomeric. LOH at the ADAR2 locus was analyzed by direct sequencing of the polymorphism rs1051367 (A/G). Green boxes represent LOH, red boxes indicate retention of heterozygosity, and yellow boxes are non-informative loci.

We studied the presence of homozygosity at 21q22.3 in genomic DNA from 48 patients with lung cancer. The evaluation of the eight microsatellites in tumor tissues and matched normal tissues allowed us to precisely determine the frequency of LOH at this chromosomal region. Figure 5A illustrates the three alternative electrophoretic patterns obtained: non-informative case, retention of heterozygosity, and LOH. After microsatellite analysis, LOH was identified in approximately 30–40% of cases (Figure 5B). LOH at 21q22.3 was significantly higher in squamous cell carcinomas than in adenocarcinomas (25±6% vs. 49±7%, p = 0.003). No differences in the frequency of LOH were found between stages (stage I: 37±2% vs. stages II–III: 34±7%, p = 0.279). To evaluate the ADAR2 locus more specifically, a SNP located within the ADAR2 gene (rs1051367) was analyzed. Of the twenty informative cases (i.e., cases heterozygous for SNP rs1051367 in normal tissue), fifteen (75%) were homozygous in the corresponding matched tumor tissue. A comparison between the results obtained from the microsatellite analysis and SNP sequencing demonstrated concordance between the respective techniques, although the number of patients with LOH at the ADAR2 SNP was higher (30–40% vs. 75%).

### Expression of RNA Metabolism-related Genes and Lung Cancer Clinical Outcome

**Figure 6:**
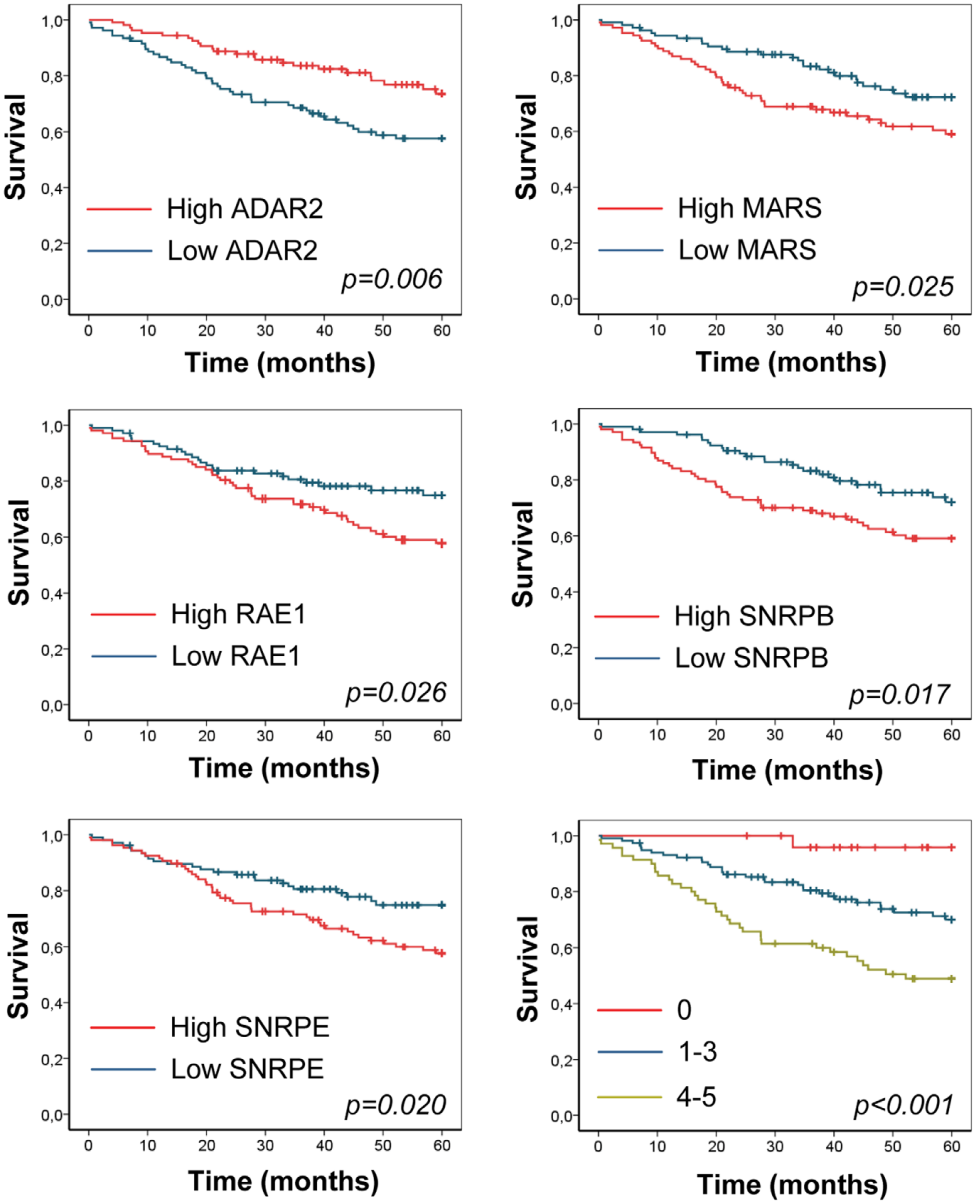
Association between RNA metabolism-related genes and clinical outcome of patients with lung adenocarcinoma. Data were obtained from a microarray study [26]. Figures show Kaplan-Meier curves and log rank statistics for overall survival in patients divided in high and low mRNA expression (using the median as the cut-off point). Last figure corresponds to patients divided by the number of deregulating events (see Material and Methods).

We investigated whether expression of the differentially expressed RNA metabolism-related genes was associated with clinical outcomes in patients with lung adenocarcinoma using a publicly available microarray data [26]. Patients were divided according to high and low mRNA expression levels using the median as the cut off point (Figure 6). In the case of ASCC3L1, MRPL3, and PABPC1, no association was found between mRNA levels and patient clinical outcome (data not shown). High expression of MARS, RAE1, SNRPB, and SNRPE was significantly associated with reduced overall survival. High ADAR2 mRNA levels were significantly associated with a better outcome. For a combined analysis of the five prognostic genes, patients were divided into three groups: patients with no deregulating events, patients with one to three events, and patients with four or five events. The combined score of the five genes was a strong prognostic marker (Figure 6). Thus, patients with no deregulating events exhibited very good survival. In contrast, patients with deregulation in four or five of the genes exhibited the worst survival. The Cox proportional hazards model was used to assess the impact of the prognostic score on overall survival, in both univariate and multivariate analysis (Table 1). The RNA-metabolism score was an independent prognostic factor for overall survival in patients with lung adenocarcinoma.

**Table 1:** Cox proportional-hazards models for association of the RNA metabolism prognostic score and the clinical outcome of patients with lung adenocarcinoma, in both univariate and multivariate analysis.

	Hazard ratio (95% CI)	p
*Univariate analysis*		
**Age**		
≤70		
>70	1.36 (0.83–2.24)	0.220
**Gender**		
Female		
Male	1.22 (0.76–1.97)	0.413
**Smoking status**		0.387
Never smoker		
Former	1.27 (0.58–2.81)	0.549
Current	1.90 (0.69–5.25)	0.214
**Stage**		<0.001
I		
II	3.07 (1.74–5.44)	<0.001
III	5.74 (3.01–10.94)	<0.001
**Differentiation grade**		
Well-differentiated		
Moderately	1.26 (0.62–2.55)	0.520
Poorly	1.51 (0.71–3.19)	0.283
**Prognostic score**		<0.001
0		
1–3	7.49 (1.02–54.85)	0.048
4–5	16.03 2.19–117.02)	0.006
*Multivariate analysis*		
**Stage**		<0.001
I		
II	2.82 (1.59–5.01)	<0.001
III	8.89 (4.47–17.35)	<0.001
**Prognostic score**		<0.001
0		
1–3	8.49 (1.15–62.44)	0.036
4–5	22.70 (3.06–168.34)	0.002

## Discussion

We identified eight RNA-related genes differentially expressed in lung cancer, a finding that supports the hypothesis that RNA metabolism is important in the pathogenesis of lung cancer. Seven of the identified genes were up-regulated, whereas only one was down-regulated. Interestingly, most of these genes had not been previously reported to be associated with lung cancer. Additionally, the expression of the majority of these genes exhibited an association with overall survival in lung adenocarcinoma patients, suggesting a connection between RNA metabolism and lung cancer pathogenesis.

Previous reports have demonstrated that RNA-metabolism genes are deregulated and implicated in malignant transformation of lung cells [14], [16], [18]. The findings in our study identify a new set of differentially-expressed genes associated with RNA-metabolism. It is clear that the expression of many other RNA-related genes is altered in lung cancer. The group of genes identified in our study was strongly influenced by the stringent strategy of selection. Statistical analyses were restrictive; thus, only the most significant genes were chosen. Moreover, changes in splicing, which have been reported for some RNA-related genes [17], [27], [28], cannot be detected using this analytical approach.

Most of the RNA-related genes identified in our study were up-regulated in tumor tissue. The reason for this is unclear; it may be due to the increased metabolic rate associated with tumor cell proliferation. However, the vast majority of RNA-related genes exhibited no differences between tumor and normal lung tissues. Moreover, one relevant gene identified in our study was downregulated (ADAR2), and mRNA expression of five of the selected genes was associated with clinical outcome in a series of adenocarcinoma patients. In particular, high expression of up-regulated genes (MARS, RAE1, SNRPB, and SNRPE) was associated with worse prognosis, whereas high expression of the downregulated gene (ADAR2) correlated with improved survival. Interestingly, the group of patients with no deregulation in any of these genes exhibited very good survival. On the other hand, a high number of deregulating events was associated with poor survival. These results suggest that the activity of the RNA-metabolic machinery could potentially serve as a prognostic marker for lung cancer.

Proteins translated from three of the eight up-regulated genes belong to the family of spliceosomal small nuclear ribonucleoproteins (snRNPs): ASCC3L1, SNRPB, and SNRPE. The spliceosome is a complex of snRNPs plus a multitude of associated proteins. This complex recognizes splice sites and removes introns from pre-mRNA molecules. Little is known regarding the role played by the spliceosome in cancer. Recently, using whole-exome sequencing analysis, some studies have identified a high frequency of mutations in distinct components of the spliceosome in chronic lymphocytic leukemia and myelodysplasia [29]–[33], implicating this cellular machinery in the development of cancer [34]. Thus, some spliceosomal inhibitors have been tested in cancer cells and the spliceosome has been proposed as an anti-cancer target [35]. The identification of three spliceosomal proteins differentially expressed in lung cancer suggests that a role is played by the spliceosome in this malignancy. Unfortunately, there is little published information concerning the role of these particular proteins in cancer. ASCC3L1 (SNRNP200) encodes helicase Brr2, an important component of the spliceosomal snRNP U5. To our knowledge, this study is the first to demonstrate than ASCC3L1 is associated with malignancy. Small nuclear ribonucleotide associated protein B (SNRPB) is part of snRNP U1. SNRPB has been reported to be a metastasis suppressor in a mouse allograft model of prostate cancer [36]. A rare polymorphism in the SNRPB gene has been associated with reduced risk of breast cancer in BRCA1 mutation carriers [37]. The overexpression of small nuclear ribonucleotide associated protein E (SNRPE) has been reportedly associated with growth arrest at G2 phase in both malignant and non-malignant cells [38]. However, in line with our results, SNRPE is amplified and overexpressed in malignant gliomas and oral squamous cell carcinomas [39], [40]. SNRPE is also amplified and up-regulated in hepatocellular carcinoma and may function as an oncogene by enhancing cell proliferation [41].

The other proteins that were up-regulated in our study were MARS, MRPL3, PABPC1 and RAE. Methionine-tRNA synthetase (MARS or MetRS) acts as a catalyst in the binding of methionine to its corresponding tRNA. Increased activity of this enzyme has been reported in human colon cancer [42]. More recently, an induction of MARS expression was shown in breast cancer cell lines stimulated with insulin-like growth factor [43]. Frameshift mutations in MARS have been described in gastric and colorectal carcinomas with microsatellite instability [44]. Mitochondrial ribosomal protein L3 (MRPL3) is a component of the 39S subunit of the mitochondrial ribosome. High expression of this protein has been reported in hepatocarcinoma, colon carcinoma, and lymphoma, suggesting an association of this protein with high cell division rates [45]. Poly-A binding protein cytoplasmic 1 (PABPC1) participates in poly-A shortening at the 3′ end of eukaryotic mRNAs. Contradictory results regarding the role of this protein in cancer have been reported. One study concluded that low levels of PABPC1 correlated with more invasive tumors and worse survival rates in patients with esophageal cancer [46]. However, in other studies, PABPC1 over-expression was described in prostate tumors [47], hepatocellular carcinoma [48], superficial bladder cancer [49], and lung cancer [50]; in the latter report the authors suggested the participation of the translation initiation complex in the tumorigenesis of lung cancer. PABPC1 also regulates telomerase activity, leading to a growth advantage in keratinocytes expressing human papillomavirus type 16 E6 [51]. RNA export 1 homolog (RAE1) is a nuclear export protein involved in mRNA transport from the nucleus to the cytoplasm. RAE1 also plays a critical role in the maintenance of spindle bipolarity during cell division [52], [53]. RAE1 mRNA and protein levels decrease upon inhibition of neuroblastoma cell proliferation, and its overexpression prevents retinoic acid-induced cell cycle arrest and differentiation [54]; however, a previous study demonstrated that RAE1/NUP98 mutant mice are more susceptible to DMBA-induced lung tumors compared to wild-type mice, indicating that combined RAE1/NUP98 haplo-insufficiency potentially promotes tumorigenesis [55].

The downregulated gene adenosine deaminase acting on RNA 2 (ADAR2 or ADARB1) is an RNA editase that catalyzes adenosine to inosine deamination in double-stranded regions. Dysregulation of adenosine to inosine editing in human cancers potentially contributes to the altered transcriptional program necessary to sustain carcinogenesis [56]. ADAR2 is ubiquitously expressed in many tissues, particularly in the central nervous system [57]. Early onset epilepsy and premature death were reported in ADAR2 knock out mice [58]. In cancer, a previous study reported overexpression of ADAR2 in *in vitro* transformed human adult mesenchymal stem cells, transformed fibroblasts, and some cell lines from other tissues [59]. Higher levels of ADAR2 mRNA were also observed in androgen-independent prostate cancer cell lines relative to androgen-responsive cell lines [60]. However, most reports link cancer with reduced ADAR2 expression or activity. Thus, a decrease in enzymatic activity of ADAR2 in patients with multiform glioblastoma (MGB) was associated with higher Ca^2+^ permeability and activation of the Akt pathway, contributing to tumor growth and aggressiveness [61], [62]. Paz et al. also found a decrease of ADAR2 mRNA levels in brain tumors and demonstrated that its overexpression in an MGB cell line resulted in decreased cell proliferation [63]. A decrease in ADAR2 editing activity, which correlated with the grade of malignancy, was also found in pediatric astrocytomas [64]. When the editing status was reverted in three astrocytoma cell lines, a significant decrease in cell malignant behavior was found [64]. More recently, Galeano et al. observed a general decrease in ADAR2-mediated editing events in bladder and colorectal cancer [65]. In the case of lung cancer, a reduction of ADAR2 expression was previously described in squamous cell lung carcinoma [66].

Downregulation of ADAR2 in lung cancer is potentially associated with genetic alterations at 21q22, where the ADAR2 gene is located. Previous studies have reported the loss of genetic material at the long arm of chromosome 21 in patients with several solid tumors [67]–[71], including lung cancer [72]–[76]. Lee et al. analyzed nine microsatellite markers, placed between 21q21.1 and 21q22.3 in NSCLC patients. LOH was detected for at least one of them in over 55% of tumors, with 26%–48% LOH incidence rate in individual microsatellites [74]. Sato et al. described LOH at 21q22.3 in 28% of samples from adenocarcinoma and squamous cell carcinoma patients [72]. We focused on the analysis of 21q22.3 alterations in the region of ADAR2. We found 30–40% incidence of LOH among patients with NSCLC, with almost half of the tumors (23 out of 48) exhibiting LOH in at least one of the microsatellites analyzed. In accordance with previous studies, squamous cell carcinomas showed higher frequencies of LOH at 21q22 than adenocarcinomas. In addition, a very high incidence of LOH (75%) was observed upon analysis of a SNP located within the ADAR2 gene. These results can be explained by the existence of alternating regions with and without LOH, and indicate that the ADAR2 genetic locus is one of the most frequently altered regions in lung carcinogenesis. Taken together, the frequent genetic losses at the ADAR2 locus and its reduced expression suggest that ADAR2 potentially functions as a tumor suppressor in lung cancer. Functional data also support this hypothesis, because overexpression of ADAR2 in cancer cell lines inhibits proliferation and migration [63], [64]. Moreover, we have demonstrated that low ADAR2 mRNA levels are significantly associated with overall survival in lung adenocarcinoma patients. A prognostic score based on the expression of ADAR2 and four additional RNA metabolism-related genes (MARS, RAE1, SNRPB and SNRPE) can stratify lung cancer patients into high and low risk groups for cancer death. Additional research is warrant to examine whether this information can be useful to identify those patients with resectable NSCLC who are at high risk of recurrence and would benefit from adjuvant therapy.

In conclusion, in this study we identified new RNA metabolism-related genes differentially expressed in lung cancer and associated with clinical outcome. These results support the role of RNA metabolism in the pathogenesis of this disease. Further characterization of the mechanisms regulating this process may potentially lead to the development of improved strategies for diagnosis, prognosis, and treatment of lung cancer.

## Materials and Methods

### Ethics Statement

This study was approved by the ethics committees of the Clínica Universidad de Navarra (Pamplona, Spain) and the Hospital Marqués de Valdecilla (Santander, Spain). Written informed consent was obtained from each patient.

### Microarray Experiments

Four publicly available microarray experiments were used to identify RNA-metabolism related genes differentially expressed between lung adenocarcinoma and normal lung tissue [22]–[25]. Some of these experiments included data from other histological subtypes. A fifth microarray experiment was used to analyze the relationship between gene expression levels and prognosis in patients with lung adenocarcinoma [26]. Table S3 contains information about the number of samples and the histological subtypes present in each study.

### Lung Cancer Cell Lines and Primary Cultures

Lung cancer cell lines were obtained from the American Type Culture Collection (ATCC, Manassas, VA). Cells were grown in RPMI supplemented with 2 mM glutamine, 10% fetal bovine serum, 100 U/ml penicillin, and 100 µg/ml streptomycin (Invitrogen, Carlsbad, CA). Small airway epithelial cells (SAEC) were purchased from Lonza (Walkersville, MD) and cultured in small airway epithelial growth medium (SAGM, Lonza) supplemented with SAGM SingleQuots (Lonza). Normal human bronchial epithelial (NHBE) cells (Clonetics, San Diego, CA) were grown in bronchial epithelial cell growth medium (BEGM, Clonetics) complemented with the growth supplements of the BulletKit (Clonetics). Cell cultures were maintained at 37°C and 5% CO_2_ in a humidified incubator. Before RNA or DNA extraction, cells were tested for *Mycoplasma* contamination, according to manufacturer’s instructions (MycoAlert Mycoplasma Detection Kit, Cambrex, Rockland, ME).

### Clinical Samples

Primary tumors and their corresponding normal lung tissues were obtained from patients with NSCLC treated with curative resectional surgery at the Clínica Universidad de Navarra (Pamplona, Spain) or at the Hospital Marqués de Valdecilla (Santander, Spain). None of the patients received chemo- or radiotherapy prior to surgery. Surgically removed samples were immediately frozen in liquid nitrogen and stored at −80°C until use. Only samples containing more than 70% tumor cells were used. Histologies and stages of the tumors included in the study are listed in Table S4. Tumors were classified according to WHO 2004 classification [77].

### RNA Extraction

RNA extraction was performed using RNA Ultraspec (Biotecx Laboratories, Houston, TX). For primary tissues, RNA extraction was carried out after mechanical fragmentation of frozen samples, using β-mercaptoethanol and RNeasy Micro (Qiagen, Hilden, Germany), according to manufacturer’s instructions. All RNA samples were diluted in water with DEPC and stored at −80°C until use. RNA concentration was determined by spectrophotometry (Nanodrop, Thermo Scientific, Wilmington, DE).

### Reverse Transcription (RT)

Two micrograms of RNA were incubated with 1 mM dNTPs and 50 ng/µL oligo-dTs at 65°C for 5 minutes. Afterwards, 4 µL of 5× RT buffer, 1 µL of 0.1 M DTT, 40 U of RNase Out and 200 U of Super Script III reverse transcriptase (all from Invitrogen) were added. Tubes were incubated 50 minutes at 50°C and 15 minutes at 70°C, and then placed into ice. Finally, 2 U of RNase H (Invitrogen) were added, and tubes were incubated 20 minutes at 37°C. cDNA was stored at −20°C until use.

### Genomic DNA Extraction

Genomic DNA was purified with the QIAamp DNA Mini Kit (Qiagen), following manufacturer’s instructions. Extraction of genomic DNA from clinical samples was performed after mechanical fragmentation using the protocol described above. DNA concentration was determined by spectrophotometry (Nanodrop).

### Conventional PCR

Gene expression was assessed by conventional PCR. GAPDH was used as control gene. Primer sequences are shown in Table S5. The reaction mixture consisted of 1 µL of cDNA, 5 µL of 10× buffer (Bioline, London, UK), 1.5 mM MgCl_2_ (Bioline), 200 µM dNTPs (Bioline), 200 nM of sense and antisense primers (Sigma, St. Louis, MO) and 2 U of Biotaq DNA Polymerase (Bioline). PCR conditions were: 2 minutes at 94°C; 30 seconds at 94°C, 30 seconds at 55°C, 30 seconds at 72°C, for 20–30 cycles (Table S5); and 10 minutes at 72°C. PCR products were separated by horizontal electrophoresis for 30 minutes at a constant voltage (100 V) in a 1% agarose gel, using SYBR Safe (Invitrogen) to visualize bands.

### Real-time PCR

Quantitative gene expression was studied by real time PCR. Primer sequences are shown in Table S6. The reaction mixture was: 0.2 µL of cDNA, 12.5 µL of SYBR Green PCR Master Mix (Applied Biosystems, Forster City, CA) and 300 nM of sense and antisense primers (Sigma). All amplifications were done in a 7300 Real Time PCR System (Applied Biosystems) using the following conditions: 2 minutes at 50°C; 10 minutes at 95°C; 15 seconds at 95°C and 1 minute at 60°C, for 40 cycles. RNA expression of each gene was normalized with HPRT expression. A tumor/normal expression ratio was calculated. In the case of the cell lines, this ratio was obtained dividing the normalized RNA expression of the gene in the cell line by its normalized expression in NHBE cells. In patient samples, the ratio was calculated dividing the normalized gene expression in the tumor tissue by the normalized gene expression in its corresponding normal tissue.

### Microsatellite Analysis

Microsatellites at 21q22.3 were analyzed to determine loss of heterozygosity (LOH). Informative microsatellites (those with maximum heterozygosity) were selected using different databases (www.ensembl.org, www.ncbi.nlm.nih.gov, and www.cephb.fr). Characteristics of the selected microsatellites are shown in Table S7. PCRs with specific fluorescence primers were done using the following reaction mixture: 20 ng of DNA, 1 µL of 10× buffer (Bioline), 2 mM MgCl_2_, 250 µM dNTPs (Bioline), 250 nM 6-FAM-labeled sense and antisense primers (Sigma), 0.5 µL of DMSO (Sigma) and 0.4 U of Biotaq DNA polymerase (Bioline). PCRs were carried out in a DNA Engine Tetrad 2 Peltier Thermal Cycler (Bio-Rad, Hercules, CA), using this program: 10 minutes at 95°C; 30 seconds at 95°C, 30 seconds at annealing temperature (Table S7), and 45 seconds at 72°C, for 40 cycles; and 10 minutes at 72°C. One microliter from a 1∶10 dilution of the amplification product was added to 20 µL of formamide and 0.2 µL of GeneScan-500 LIZ Size Standard (Applied Biosystems). The mixture was separated by capillary electrophoresis in a 3130xl Genetic Analyzer and data were analyzed by Gene Mapper Software 3.7 (both from Applied Biosystems). LOH was determined by this ratio: (N_1_/N_2_)/(T_1_/T_2_), where N_1_ and N_2_ represent the areas of the two alleles peaks in the normal sample and T_1_ and T_2_ are the areas of the allele peaks in the corresponding tumor sample. LOH was considered present when the ratio was <0.5 or >2.

### SNP Analysis

A single nucleotide polymorphism (SNP) located within the ADAR2 gene (rs1051367) was analyzed by PCR. Primers used to amplify the region containing this SNP were: sense, 5′-CTTCCTCTGGGTTGCTTTC-3′; antisense, 5′-TCAGGGCGTGAGTGAG-3′. One hundred nanograms of genomic DNA were used to run a 30-cycle PCR. Reaction conditions were the same as those described above for conventional PCR. The amplification product was sequenced by capillary electrophoresis using BigDye Terminator 3.1 in a 3130xl Genetic Analyzer (Applied Biosystems). Sequences were analyzed using Chromas Lite 2.01 (Technelysium, Brisbane, Australia).

### Statistical Analysis

Significant differences in expression levels of RNA metabolism-related genes were analyzed by ANOVA. LOH frequencies were compared using the Mann-Whitney U-test. Kaplan-Meier plots were used to illustrate differences in progression according to the mRNA levels of the selected genes. mRNA expression data were obtained from an extensive study of lung adenocarcinomas [26]. Patients with adjuvant chemo- or radiotherapy were excluded. Information about overall survival and gene expression was available from 213 patients. Clinicopathological features of these patients are shown in Table S8. Expression was dichotomized using the median value. Overall survival (censored at 60 months) was used as the outcome variable. Probesets used were: 203865_s_at (ADAR2), 200058_s_at (ASCC3L1), 201475_s_at (MARS), 208787_s_at (MRPL3), 215157_s_at (PABPC1), 211318_s_at (RAE1), 208821_s_at (SNRPB), 203316_s_at (SNRPE). For the combined analysis of prognostic genes, patients were divided into three groups: patients with no deregulating events, patients with one to three events, and patients with four or five events. A deregulating event was defined by a high expression of an up-regulated gene (MARS, RAE1, SNRPB or SNRPE) or a low expression of the donwregulated one (ADAR2). Significant differences in survival were analyzed using the log-rank test. Suvival was correlated with age, gender, smoking status, stage, differentiation grade and the prognostic score in a univariate Cox proportional hazards analysis. Multivariate analysis was performed on parameters found to be significant on univariate analysis. p-values <0.05 were considered statistically significant.

## Supporting Information

Table S1 RNA metabolism-related genes with significant differences in its expression between lung adenocarcinoma and normal lung tissue.
(PDF)

Table S2 Statistical validation of selected genes using microarray data from a fourth cohort of lung cancer patients (Yap et al., 2005).
(PDF)

Table S3 Number of cases and histological types in the microarray experiments used in the study.
(PDF)

Table S4 Pathological characteristics of the primary tumors.
(PDF)

Table S5 Primers used for conventional PCR.
(PDF)

Table S6 Primers used for real-time PCR.
(PDF)

Table S7 Microsatellites analyzed at 21q22.3.
(PDF)

Table S8 Demographic and clinical characteristics of the 213 patients selected from the cohort of adenocarcinoma patients (Shedden et al., 2008).
(PDF)

## References

[1] Kohler BA, Ward E, McCarthy BJ, Schymura MJ, Ries LA, et al (2011) Annual report to the nation on the status of cancer, 1975–2007, featuring tumors of the brain and other nervous system. J Natl Cancer Inst 103: 714–736. 21454908 10.1093/jnci/djr077 PMC3086878

[2] Ferlay J, Parkin DM, Steliarova-Foucher E (2010) Estimates of cancer incidence and mortality in Europe in 2008. Eur J Cancer 46: 765–781. 20116997 10.1016/j.ejca.2009.12.014

[3] David CJ, Manley JL (2010) Alternative pre-mRNA splicing regulation in cancer: pathways and programs unhinged. Genes Dev 24: 2343–2364. 21041405 10.1101/gad.1973010 PMC2964746

[4] Pajares MJ, Ezponda T, Catena R, Calvo A, Pio R, et al (2007) Alternative splicing: an emerging topic in molecular and clinical oncology. Lancet Oncol 8: 349–357. 17395108 10.1016/S1470-2045(07)70104-3

[5] Xi L, Feber A, Gupta V, Wu M, Bergemann AD, et al (2008) Whole genome exon arrays identify differential expression of alternatively spliced, cancer-related genes in lung cancer. Nucleic Acids Res 36: 6535–6547. 18927117 10.1093/nar/gkn697 PMC2582617

[6] Pio R, Montuenga LM (2009) Alternative splicing in lung cancer. J Thorac Oncol 4: 674–678. 19461400 10.1097/JTO.0b013e3181a520dc

[7] Langer W, Sohler F, Leder G, Beckmann G, Seidel H, et al (2010) Exon array analysis using re-defined probe sets results in reliable identification of alternatively spliced genes in non-small cell lung cancer. BMC Genomics 11: 676. 21118496 10.1186/1471-2164-11-676 PMC3053589

[8] Pio R, Blanco D, Pajares MJ, Aibar E, Durany O, et al (2010) Development of a novel splice array platform and its application in the identification of alternative splice variants in lung cancer. BMC Genomics 11: 352. 20525254 10.1186/1471-2164-11-352 PMC2889901

[9] Misquitta-Ali CM, Cheng E, O'Hanlon D, Liu N, McGlade CJ, et al (2011) Global profiling and molecular characterization of alternative splicing events misregulated in lung cancer. Mol Cell Biol 31: 138–150. 21041478 10.1128/MCB.00709-10 PMC3019846

[10] Groeger AM, Esposito V, De Luca A, Cassandro R, Tonini G, et al (2004) Prognostic value of immunohistochemical expression of p53, bax, Bcl-2 and Bcl-xL in resected non-small-cell lung cancers. Histopathology 44: 54–63. 14717670 10.1111/j.1365-2559.2004.01750.x

[11] Karczmarek-Borowska B, Filip A, Wojcierowski J, Smolen A, Korobowicz E, et al (2006) Estimation of prognostic value of Bcl-xL gene expression in non-small cell lung cancer. Lung Cancer 51: 61–69. 16297499 10.1016/j.lungcan.2005.08.010

[12] Shultz JC, Goehe RW, Murudkar CS, Wijesinghe DS, Mayton EK, et al (2011) SRSF1 regulates the alternative splicing of caspase 9 via a novel intronic splicing enhancer affecting the chemotherapeutic sensitivity of non-small cell lung cancer cells. Mol Cancer Res 9: 889–900. 21622622 10.1158/1541-7786.MCR-11-0061 PMC3140550

[13] Kong-Beltran M, Seshagiri S, Zha J, Zhu W, Bhawe K, et al (2006) Somatic mutations lead to an oncogenic deletion of met in lung cancer. Cancer Res 66: 283–289. 16397241 10.1158/0008-5472.CAN-05-2749

[14] Karni R, de Stanchina E, Lowe SW, Sinha R, Mu D, et al (2007) The gene encoding the splicing factor SF2/ASF is a proto-oncogene. Nat Struct Mol Biol 14: 185–193. 17310252 10.1038/nsmb1209 PMC4595851

[15] Pino I, Pio R, Toledo G, Zabalegui N, Vicent S, et al (2003) Altered patterns of expression of members of the heterogeneous nuclear ribonucleoprotein (hnRNP) family in lung cancer. Lung Cancer 41: 131–143. 12871776 10.1016/s0169-5002(03)00193-4

[16] Tockman MS, Mulshine JL, Piantadosi S, Erozan YS, Gupta PK, et al (1997) Prospective detection of preclinical lung cancer: results from two studies of heterogeneous nuclear ribonucleoprotein A2/B1 overexpression. Clin Cancer Res 3: 2237–2246. 9815620

[17] Castano Z, Vergara-Irigaray N, Pajares MJ, Montuenga LM, Pio R (2008) Expression of alpha CP-4 inhibits cell cycle progression and suppresses tumorigenicity of lung cancer cells. Int J Cancer 122: 1512–1520. 17973258 10.1002/ijc.23236

[18] Ezponda T, Pajares MJ, Agorreta J, Echeveste JI, Lopez-Picazo JM, et al (2010) The oncoprotein SF2/ASF promotes non-small cell lung cancer survival by enhancing survivin expression. Clin Cancer Res 16: 4113–4125. 20682707 10.1158/1078-0432.CCR-10-0076

[19] Goehe RW, Shultz JC, Murudkar C, Usanovic S, Lamour NF, et al (2010) hnRNP L regulates the tumorigenic capacity of lung cancer xenografts in mice via caspase-9 pre-mRNA processing. J Clin Invest 120: 3923–3939. 20972334 10.1172/JCI43552 PMC2964989

[20] Zerbe LK, Pino I, Pio R, Cosper PF, Dwyer-Nield LD, et al (2004) Relative amounts of antagonistic splicing factors, hnRNP A1 and ASF/SF2, change during neoplastic lung growth: implications for pre-mRNA processing. Mol Carcinog 41: 187–196. 15390079 10.1002/mc.20053

[21] Peebles KA, Dwyer-Nield LD, Malkinson AM (2007) Altered expression of splicing factor, heterogeneous nuclear ribonucleoprotein A2/B1, in mouse lung neoplasia. Mol Carcinog. 2007/05/05 ed. 887–900. 10.1002/mc.20321 17477362

[22] Bhattacharjee A, Richards WG, Staunton J, Li C, Monti S, et al (2001) Classification of human lung carcinomas by mRNA expression profiling reveals distinct adenocarcinoma subclasses. Proc Natl Acad Sci U S A 98: 13790–13795. 11707567 10.1073/pnas.191502998 PMC61120

[23] Beer DG, Kardia SL, Huang CC, Giordano TJ, Levin AM, et al (2002) Gene-expression profiles predict survival of patients with lung adenocarcinoma. Nat Med 8: 816–824. 12118244 10.1038/nm733

[24] Dehan E, Ben-Dor A, Liao W, Lipson D, Frimer H, et al (2007) Chromosomal aberrations and gene expression profiles in non-small cell lung cancer. Lung Cancer 56: 175–184. 17258348 10.1016/j.lungcan.2006.12.010

[25] Yap YL, Lam DC, Luc G, Zhang XW, Hernandez D, et al (2005) Conserved transcription factor binding sites of cancer markers derived from primary lung adenocarcinoma microarrays. Nucleic Acids Res 33: 409–421. 15653641 10.1093/nar/gki188 PMC546166

[26] Shedden K, Taylor JM, Enkemann SA, Tsao MS, Yeatman TJ, et al (2008) Gene expression-based survival prediction in lung adenocarcinoma: a multi-site, blinded validation study. Nat Med 14: 822–827. 18641660 10.1038/nm.1790 PMC2667337

[27] Sueoka E, Sueoka N, Iwanaga K, Sato A, Suga K, et al (2005) Detection of plasma hnRNP B1 mRNA, a new cancer biomarker, in lung cancer patients by quantitative real-time polymerase chain reaction. Lung Cancer 48: 77–83. 15777973 10.1016/j.lungcan.2004.10.007

[28] Pio R, Zudaire I, Pino I, Castano Z, Zabalegui N, et al (2004) Alpha CP-4, encoded by a putative tumor suppressor gene at 3p21, but not its alternative splice variant alpha CP-4a, is underexpressed in lung cancer. Cancer Res 64: 4171–4179. 15205328 10.1158/0008-5472.CAN-03-2982

[29] Yoshida K, Sanada M, Shiraishi Y, Nowak D, Nagata Y, et al (2011) Frequent pathway mutations of splicing machinery in myelodysplasia. Nature 478: 64–69. 21909114 10.1038/nature10496

[30] Abdel-Wahab O, Levine R (2011) The spliceosome as an indicted conspirator in myeloid malignancies. Cancer Cell 20: 420–423. 22014568 10.1016/j.ccr.2011.10.004 PMC3218079

[31] Papaemmanuil E, Cazzola M, Boultwood J, Malcovati L, Vyas P, et al (2011) Somatic SF3B1 mutation in myelodysplasia with ring sideroblasts. N Engl J Med 365: 1384–1395. 21995386 10.1056/NEJMoa1103283 PMC3322589

[32] Quesada V, Conde L, Villamor N, Ordoñez GR, Jares P, et al (2011) Exome sequencing identifies recurrent mutations of the splicing factor SF3B1 gene in chronic lymphocytic leukemia. Nat Genet 44: 47–52. 22158541 10.1038/ng.1032

[33] Wang L, Lawrence MS, Wan Y, Stojanov P, Sougnez C, et al (2011) SF3B1 and other novel cancer genes in chronic lymphocytic leukemia. N Engl J Med 365: 2497–2506. 22150006 10.1056/NEJMoa1109016 PMC3685413

[34] Ebert B, Bernard OA (2011) Mutations in RNA splicing machinery in human cancers. N Engl J Med 365: 2534–2535. 22150007 10.1056/NEJMe1111584

[35] van Alphen RJ, Wiemer EA, Burger H, Eskens FA (2009) The spliceosome as target for anticancer treatment. Br J Cancer 100: 228–232. 19034274 10.1038/sj.bjc.6604801 PMC2634708

[36] Yi Y, Nandana S, Case T, Nelson C, Radmilovic T, et al (2009) Candidate metastasis suppressor genes uncovered by array comparative genomic hybridization in a mouse allograft model of prostate cancer. Mol Cytogenet 2: 18. 19781100 10.1186/1755-8166-2-18 PMC2761934

[37] Wang X, Pankratz VS, Fredericksen Z, Tarrell R, Karaus M, et al (2010) Common variants associated with breast cancer in genome-wide association studies are modifiers of breast cancer risk in BRCA1 and BRCA2 mutation carriers. Hum Mol Genet 19: 2886–2897. 20418484 10.1093/hmg/ddq174 PMC2893806

[38] Li Z, Putzer BM (2008) Spliceosomal protein E regulates neoplastic cell growth by modulating expression of cyclin E/CDK2 and G2/M checkpoint proteins. J Cell Mol Med 12: 2427–2438. 18208561 10.1111/j.1582-4934.2008.00244.x PMC4514120

[39] Riemenschneider MJ, Knobbe CB, Reifenberger G (2003) Refined mapping of 1q32 amplicons in malignant gliomas confirms MDM4 as the main amplification target. Int J Cancer 104: 752–757. 12640683 10.1002/ijc.11023

[40] Kuo WP, Jenssen TK, Park PJ, Lingen MW, Hasina R, et al (2002) Gene expression levels in different stages of progression in oral squamous cell carcinoma. Proc AMIA Symp. 415–419. 12474876 PMC2244435

[41] Jia D, Wei L, Guo W, Zha R, Bao M, et al (2011) Genome-wide copy number analyses identified novel cancer genes in hepatocellular carcinoma. Hepatology. 10.1002/hep.24495 21688285

[42] Kushner JP, Boll D, Quagliana J, Dickman S (1976) Elevated methionine-tRNA synthetase activity in human colon cancer. Proc Soc Exp Biol Med 153: 273–276. 995958 10.3181/00379727-153-39526

[43] Pacher M, Seewald MJ, Mikula M, Oehler S, Mogg M, et al (2007) Impact of constitutive IGF1/IGF2 stimulation on the transcriptional program of human breast cancer cells. Carcinogenesis 28: 49–59. 16774935 10.1093/carcin/bgl091

[44] Park SW, Kim SS, Yoo NJ, Lee SH (2010) Frameshift Mutation of MARS Gene Encoding an Aminoacyl-tRNA Synthetase in Gastric and Colorectal Carcinomas with Microsatellite Instability. Gut Liver 4: 430–431. 20981229 10.5009/gnl.2010.4.3.430 PMC2956364

[45] Ou JH, Yen TS, Wang YF, Kam WK, Rutter WJ (1987) Cloning and characterization of a human ribosomal protein gene with enhanced expression in fetal and neoplastic cells. Nucleic Acids Res 15: 8919–8934. 2891103 10.1093/nar/15.21.8919 PMC306413

[46] Takashima N, Ishiguro H, Kuwabara Y, Kimura M, Haruki N, et al (2006) Expression and prognostic roles of PABPC1 in esophageal cancer: correlation with tumor progression and postoperative survival. Oncol Rep 15: 667–671. 16465428

[47] van Duin M, van Marion R, Vissers K, Watson JE, van Weerden WM, et al (2005) High-resolution array comparative genomic hybridization of chromosome arm 8q: evaluation of genetic progression markers for prostate cancer. Genes Chromosomes Cancer 44: 438–449. 16130124 10.1002/gcc.20259

[48] Liu Y, Zhu X, Zhu J, Liao S, Tang Q, et al (2007) Identification of differential expression of genes in hepatocellular carcinoma by suppression subtractive hybridization combined cDNA microarray. Oncol Rep 18: 943–951. 17786358

[49] Chen R, Feng C, Xu Y (2011) Cyclin-dependent kinase-associated protein Cks2 is associated with bladder cancer progression. J Int Med Res 39: 533–540. 21672358 10.1177/147323001103900222

[50] Comtesse N, Keller A, Diesinger I, Bauer C, Kayser K, et al (2007) Frequent overexpression of the genes FXR1, CLAPM1 and EIF4G located on amplicon 3q26–27 in squamous cell carcinoma of the lung. Int J Cancer 120: 2538–2544. 17290396 10.1002/ijc.22585

[51] Katzenellenbogen RA, Vliet-Gregg P, Xu M, Galloway DA (2010) Cytoplasmic poly(A) binding proteins regulate telomerase activity and cell growth in human papillomavirus type 16 E6-expressing keratinocytes. J Virol 84: 12934–12944. 20943973 10.1128/JVI.01377-10 PMC3004306

[52] Wong RW (2010) Interaction between Rae1 and cohesin subunit SMC1 is required for proper spindle formation. Cell Cycle 9: 198–200. 20016259 10.4161/cc.9.1.10431

[53] Wong RW, Blobel G, Coutavas E (2006) Rae1 interaction with NuMA is required for bipolar spindle formation. Proc Natl Acad Sci U S A 103: 19783–19787. 17172455 10.1073/pnas.0609582104 PMC1750899

[54] Cuende J, Moreno S, Bolanos JP, Almeida A (2008) Retinoic acid downregulates Rae1 leading to APC(Cdh1) activation and neuroblastoma SH-SY5Y differentiation. Oncogene 27: 3339–3344. 18212744 10.1038/sj.onc.1210987

[55] Jeganathan KB, Baker DJ, van Deursen JM (2006) Securin associates with APCCdh1 in prometaphase but its destruction is delayed by Rae1 and Nup98 until the metaphase/anaphase transition. Cell Cycle 5: 366–370. 16479161 10.4161/cc.5.4.2483

[56] Miura K, Fujibuchi W, Sasaki I (2011) Alternative pre-mRNA splicing in digestive tract malignancy. Cancer Sci 102: 309–316. 21134075 10.1111/j.1349-7006.2010.01797.x

[57] Melcher T, Maas S, Herb A, Sprengel R, Higuchi M, et al (1996) RED2, a brain-specific member of the RNA-specific adenosine deaminase family. J Biol Chem 271: 31795–31798. 8943218 10.1074/jbc.271.50.31795

[58] Higuchi M, Maas S, Single FN, Hartner J, Rozov A, et al (2000) Point mutation in an AMPA receptor gene rescues lethality in mice deficient in the RNA-editing enzyme ADAR2. Nature 406: 78–81. 10894545 10.1038/35017558

[59] Flanagan JM, Funes JM, Henderson S, Wild L, Carey N, et al (2009) Genomics screen in transformed stem cells reveals RNASEH2A, PPAP2C, and ADARB1 as putative anticancer drug targets. Mol Cancer Ther 8: 249–260. 19139135 10.1158/1535-7163.MCT-08-0636

[60] Martinez HD, Jasavala RJ, Hinkson I, Fitzgerald LD, Trimmer JS, et al (2008) RNA editing of androgen receptor gene transcripts in prostate cancer cells. J Biol Chem 283: 29938–29949. 18708348 10.1074/jbc.M800534200 PMC2662061

[61] Maas S, Patt S, Schrey M, Rich A (2001) Underediting of glutamate receptor GluR-B mRNA in malignant gliomas. Proc Natl Acad Sci U S A 98: 14687–14692. 11717408 10.1073/pnas.251531398 PMC64742

[62] Ishiuchi S, Yoshida Y, Sugawara K, Aihara M, Ohtani T, et al (2007) Ca2+-permeable AMPA receptors regulate growth of human glioblastoma via Akt activation. J Neurosci 27: 7987–8001. 17652589 10.1523/JNEUROSCI.2180-07.2007 PMC6672718

[63] Paz N, Levanon EY, Amariglio N, Heimberger AB, Ram Z, et al (2007) Altered adenosine-to-inosine RNA editing in human cancer. Genome Res 17: 1586–1595. 17908822 10.1101/gr.6493107 PMC2045141

[64] Cenci C, Barzotti R, Galeano F, Corbelli S, Rota R, et al (2008) Down-regulation of RNA editing in pediatric astrocytomas: ADAR2 editing activity inhibits cell migration and proliferation. J Biol Chem 283: 7251–7260. 18178553 10.1074/jbc.M708316200

[65] Galeano F, Leroy A, Rossetti C, Gromova I, Gautier P, et al (2010) Human BLCAP transcript: new editing events in normal and cancerous tissues. Int J Cancer 127: 127–137. 19908260 10.1002/ijc.25022 PMC2958456

[66] Inamura K, Fujiwara T, Hoshida Y, Isagawa T, Jones MH, et al (2005) Two subclasses of lung squamous cell carcinoma with different gene expression profiles and prognosis identified by hierarchical clustering and non-negative matrix factorization. Oncogene 24: 7105–7113. 16007138 10.1038/sj.onc.1208858

[67] Cliby W, Ritland S, Hartmann L, Dodson M, Halling KC, et al (1993) Human epithelial ovarian cancer allelotype. Cancer Res 53: 2393–2398. 8485726

[68] Sakata K, Tamura G, Nishizuka S, Maesawa C, Suzuki Y, et al (1997) Commonly deleted regions on the long arm of chromosome 21 in differentiated adenocarcinoma of the stomach. Genes Chromosomes Cancer 18: 318–321. 9087574

[69] Ohgaki K, Iida A, Kasumi F, Sakamoto G, Akimoto M, et al (1998) Mapping of a new target region of allelic loss to a 6-cM interval at 21q21 in primary breast cancers. Genes Chromosomes Cancer 23: 244–247. 9790505

[70] Yamamoto N, Uzawa K, Miya T, Watanabe T, Yokoe H, et al (1999) Frequent allelic loss/imbalance on the long arm of chromosome 21 in oral cancer: evidence for three discrete tumor suppressor gene loci. Oncol Rep 6: 1223–1227. 10523685 10.3892/or.6.6.1223

[71] Mayama T, Fukushige S, Shineha R, Nishihira T, Satomi S, et al (2000) Frequent loss of copy number on the long arm of chromosome 21 in human esophageal squamous cell carcinoma. Int J Oncol 17: 245–252. 10891531

[72] Sato S, Nakamura Y, Tsuchiya E (1994) Difference of allelotype between squamous cell carcinoma and adenocarcinoma of the lung. Cancer Res 54: 5652–5655. 7923212

[73] Kohno T, Kawanishi M, Matsuda S, Ichikawa H, Takada M, et al (1998) Homozygous deletion and frequent allelic loss of the 21q11.1–q21.1 region including the ANA gene in human lung carcinoma. Genes Chromosomes Cancer 21: 236–243. 9523199 10.1002/(sici)1098-2264(199803)21:3<236::aid-gcc8>3.0.co;2-0

[74] Lee EB, Park TI, Park SH, Park JY (2003) Loss of heterozygosity on the long arm of chromosome 21 in non-small cell lung cancer. Ann Thorac Surg 75: 1597–1600. 12735585 10.1016/s0003-4975(02)04902-0

[75] Tseng RC, Chang JW, Hsien FJ, Chang YH, Hsiao CF, et al (2005) Genomewide loss of heterozygosity and its clinical associations in non small cell lung cancer. Int J Cancer 117: 241–247. 15900585 10.1002/ijc.21178

[76] Petersen S, Aninat-Meyer M, Schluns K, Gellert K, Dietel M, et al (2000) Chromosomal alterations in the clonal evolution to the metastatic stage of squamous cell carcinomas of the lung. Br J Cancer 82: 65–73. 10638968 10.1054/bjoc.1999.0878 PMC2363206

[77] Travis WD, Brambilla E, Müller-Hermelink HK, Harris CC (2004) Pathology and Genetics of Tumours of the Lung, Pleura, Thymus and Heart. World Health Organization Classification of tumours. 3rd ed. Lyon: IARC Press.

