# Supplementary material for: Identification of Novel Deregulated RNA Metabolism-Related Genes in Non-Small Cell Lung Cancer
Source: PLoS One. 2012 Aug 2;7(8):e42086. doi: 10.1371/journal.pone.0042086 (PMC3410905; doi:10.1371/journal.pone.0042086)
Supplement: Table S1 — RNA metabolism-related genes with significant differences in its expression between lung adenocarcinoma and normal lung tissue. (PDF) [file pone.0042086.s001.pdf]

Table S1. RNA metabolism-related genes with significant differences in its expression between lung adenocarcinoma and normal lung tissue.

	p value			Fold-change		
**Gene**	**Harvard**	**Michigan**	**Tel Aviv**	**Harvard**	**Michigan**	**Tel Aviv**
**ADAR2**	< 0.001	< 0.001	0.004	-2.037	-1.851	-2.054
**ASCC3L1**	< 0.001	0.001	0.003	0.526	0.743	0.955
**MARS**	< 0.001	0.009	0.009	0.712	0.504	0.384
**MRPL3**	< 0.001	< 0.001	0.009	1.812	0.826	1.044
**PABPC1**	< 0.001	< 0.001	0.009	0.551	1.099	0.554
**RAE1**	< 0.001	0.005	0.005	0.735	0.536	1.155
**RNPS1**	0.001	0.007	< 0.001	0.365	0.418	0.922
**SNRPB**	0.007	< 0.001	0.003	0.411	0.757	0.699
**SNRPC**	0.002	0.004	0.001	0.987	1.992	1.009
**SNRPE**	< 0.001	<0.001	0.001	0.815	1.067	1.272

Significance of the ANOVA test and mean fold-change (FC) of the representing probes in the adenocarcinoma cases are shown. FC was calculated by the formula: $FC = \log_2 (T/N)$*, where T and N denote non-normalized mean gene expression in tumor and normal samples, respectively.*
