# Supplementary material for: Identification of Novel Deregulated RNA Metabolism-Related Genes in Non-Small Cell Lung Cancer
Source: PLoS One. 2012 Aug 2;7(8):e42086. doi: 10.1371/journal.pone.0042086 (PMC3410905; doi:10.1371/journal.pone.0042086)
Supplement: Table S2 — Statistical validation of selected genes using microarray data from a fourth cohort of lung cancer patients (Yap et al., 2005). (PDF) [file pone.0042086.s002.pdf]

Table S2. Statistical validation of selected genes using microarray data from a fourth cohort of lung cancer patients (Yap et al., 2005).

Gene	p value	*Fold-change*
**ADAR2**	<0.001	-1.179
**ASCC3L1**	<0.001	1.066
**MARS**	0.006	1.147
**MRPL3**	<0.001	1.852
**PABPC1**	<0.001	1.455
**RAE1**	0.002	0.937
**RNPS1**	<0.001	1.278
**SNRPB**	0.002	1.269
**SNRPC**	0.005	0.925
**SNRPE**	0.004	1.028

Significance of the ANOVA test and fold-change (FC) of the representing probes in the adenocarcinoma cases are shown. FC was calculated by the formula: FC = log2 (T/N), where T and N denote mean gene expression in tumor and normal samples, respectively.
