# Supplementary material for: Identification of Novel Deregulated RNA Metabolism-Related Genes in Non-Small Cell Lung Cancer
Source: PLoS One. 2012 Aug 2;7(8):e42086. doi: 10.1371/journal.pone.0042086 (PMC3410905; doi:10.1371/journal.pone.0042086)
Supplement: Table S3 — Number of cases and histological types in the microarray experiments used in the study. (PDF) [file pone.0042086.s003.pdf]

Table S3. Number of cases and histological types in the microarray experiments used in the study.

Database	Normal	Adenocarcinoma	Squamous	SCLC	Carcinoid
Harvard (Bhattacharjee et al., 2001)	17	139	21	6	20
Michigan (Beer et al., 2001)	10	86	-	-	-
Tel Aviv (Dehan et al., 2007)	9	7	17	-	-
Hong Kong (Yap et al., 2005)	9	49	-	-	-
Multicenter consortium (Shedden et al., 2008)	-	442	-	-	-
