# Supplementary material for: Identification of Novel Deregulated RNA Metabolism-Related Genes in Non-Small Cell Lung Cancer
Source: PLoS One. 2012 Aug 2;7(8):e42086. doi: 10.1371/journal.pone.0042086 (PMC3410905; doi:10.1371/journal.pone.0042086)
Supplement: Table S4 — Pathological characteristics of the primary tumors. (PDF) [file pone.0042086.s004.pdf]

Table S4. Pathological characteristics of the primary tumors.

Patient	Histology	Stage	Patient	Histology	Stage
1	Squamous cell carcinoma	IB	25	Squamous cell carcinoma	IA
2	Squamous cell carcinoma	IIB	26	Squamous cell carcinoma	IA
3	Squamous cell carcinoma	IB	27	Adenocarcinoma	IB
4	Adenocarcinoma	IIIA	28	Adenocarcinoma	IA
5	Squamous cell carcinoma	IB	29	Adenocarcinoma	IB
6	Squamous cell carcinoma	IB	30	Squamous cell carcinoma	IIB
7	Squamous cell carcinoma	IB	31	Squamous cell carcinoma	IIIA
8	Squamous cell carcinoma	IIIA	32	Squamous cell carcinoma	IIA
9	Squamous cell carcinoma	IIB	33	Squamous cell carcinoma	IA
10	Adenocarcinoma	IA	34	Mixed	IIB
11	Squamous cell carcinoma	IB	35	Squamous cell carcinoma	IIB
12	Squamous cell carcinoma	IB	36	Squamous cell carcinoma	IB
13	Mixed	IB	37	Squamous cell carcinoma	IB
14	Squamous cell carcinoma	IIB	38	Squamous cell carcinoma	IB
15	Adenocarcinoma	IIB	39	Adenocarcinoma	IB
16	Adenocarcinoma	IIA	40	Adenocarcinoma	IIB
17	Adenocarcinoma	IA	41	Adenocarcinoma	IA
18	Squamous cell carcinoma	IB	42	Adenocarcinoma	IB
19	Adenocarcinoma	IB	43	Adenocarcinoma	IIIA
20	Large cell carcinoma	IIB	44	Adenocarcinoma	IIB
21	Adenocarcinoma	IIA	45	Adenocarcinoma	IIIA
22	Squamous cell carcinoma	IB	46	Adenocarcinoma	IIB
23	Adenocarcinoma	IIIB	47	Adenocarcinoma	IB
24	Squamous cell carcinoma	IB	48	Adenocarcinoma	IB
