# Supplementary material for: Identification of Novel Deregulated RNA Metabolism-Related Genes in Non-Small Cell Lung Cancer
Source: PLoS One. 2012 Aug 2;7(8):e42086. doi: 10.1371/journal.pone.0042086 (PMC3410905; doi:10.1371/journal.pone.0042086)
Supplement: Table S5 — Primers used for conventional PCR. (PDF) [file pone.0042086.s005.pdf]

Table S5. Primers used for conventional PCR.

Gene	Primer	Sequence (5´→ 3´)	Position	Accession number	Cycles
**ADAR2**	S	CACCTCTCTACACCCTCAACAAG	2245-2267	gi:75709170	30
	AS	CGTAGTAAGTGGGAGGGAACCTT	2457-2479		
**ASCC3L1**	S	GTGTCTGCTCTGAGAAACAGTGC	4011-4033	gi:40217846	25
	AS	CTGTCAGGAGTACCACCTTCTTG	4292-4314		
**GAPDH**	S	ACTTTGTCAAGCTCATTTCC	1019-1038	gi:83641890	25
	AS	CACAGGGTACTTTATTGATG	1284-1303		
**MARS**	S	CTCCAGCACTATCACCAGCTACT	2088-2110	gi:14043021	20
	AS	GTGTCCTGCTGGTAAGGTACACA	2380-2402		
**MRPL3**	S	GCCTATACATCCTTGGGAACCAG	419-441	gi:21265090	25
	AS	GTGAGCAGCATAAAGAGGAGTGC	733-755		
**PABPC1**	S	CAGGCTCACCTCACTAACCAGTA	1627-1649	gi:56676313	20
	AS	GAACCTGTGAAGAAGCTGGTCTC	1902-1924		
**RAE1**	S	ATGACTGGGAGCTGGGATAAGAC	798-820	gi:62739174	25
	AS	AGCAACTCTCCCCTCGATACTTC	1066-1088		
**RNPS1**	S	TAGGGCTCCTTCACCTACCAAAC	320-342	gi:18379335	25
	AS	AAGGACTTGGAGAGCCAGAAGAG	608-630		
**SNRPB**	S	CAGAAAGGGAAGAGAAGCGAGTC	339-361	gi:38150006	20
	AS	GTACCTCTTCCTTGTGGGGTCAT	587-609		
**SNRPC**	S	CTACTCCATTCTCTGCTCCTCCT	206-228	gi:4507126	25
	AS	CCTCCCCTCTATCCTTATCTGTC	487-509		
**SNRPE**	S	TAGATCGCGGATTCAGGTG	129-147	gi:61098048	25
	AS	AGCATGATCCGACCCAGTT	266-284		

Sense (S) and antisense (AS) primer sequences are shown. Their position in the transcript, the accession number of the transcript, and the number of cycles used in each PCR are also shown.
