# Supplementary material for: Identification of Novel Deregulated RNA Metabolism-Related Genes in Non-Small Cell Lung Cancer
Source: PLoS One. 2012 Aug 2;7(8):e42086. doi: 10.1371/journal.pone.0042086 (PMC3410905; doi:10.1371/journal.pone.0042086)
Supplement: Table S6 — Primers used for real-time PCR. (PDF) [file pone.0042086.s006.pdf]

Table S6. Primers used for real-time PCR.

Gene	Primer	Sequence (5´→ 3´)	Position	Accession number
**ADAR2**	S	GTACCAGCGGATCTCCAACATAG	2213-2235	gi:75709170
	AS	CTACCGTCCAGTTGACACTGAAG	2324-2346	
**ASCC3L1**	S	CTCATCTCCATCAAGAGGCTGAC	6312-6334	gi:40217846
	AS	CTCCTGGTCACATCCCATGTAAG	6421-6443	
**HPRT**	S	TGACACTGGCAAAACAATGCA	578-598	gi:164518913
	AS	GGTCCTTTTCACCAGCAAGCT	651-671	
**MARS**	S	TACACAGACCAGTGGGAGAGATG	1617-1639	gi:14043021
	AS	GAGGTGGCTGACCAAGGTATAGT	1744-1766	
**MRPL3**	S	TGCTGCTCACTTTCGTCCA	746-764	gi:21265090
	AS	TCGTTTGACCATGCGTAGC	849-867	
**PABPC1**	S	CAACCCTGTAATCAACCCCTACC	1683-1705	gi:56676313
	AS	GCGAGGACTTGGTCTTAGTTGAG	1790-1812	
**RAE1**	S	CCTCAGCAGTAACCAAGCGATAC	707-729	gi:62739174
	AS	GTCTTATCCCAGCTCCCAGTCAT	798-820	
**SNRPB**	S	CAGAAAGGGAAGAGAAGCGAGTC	339-361	gi:38150006
	AS	TCCAGCAAGTGGAACTCGAGCAA	438-460	
**SNRPE**	S	TAGATCGCGGATTCAGGTG	129-147	gi:61098048
	AS	AGCATGATCCGACCCAGTT	266-284	

Sense (S) and antisense (AS) primer sequences are shown, as well as their position in the transcript and its accession number.
