# Supplementary material for: Identification of Novel Deregulated RNA Metabolism-Related Genes in Non-Small Cell Lung Cancer
Source: PLoS One. 2012 Aug 2;7(8):e42086. doi: 10.1371/journal.pone.0042086 (PMC3410905; doi:10.1371/journal.pone.0042086)
Supplement: Table S7 — Microsatellites analyzed at 21q22.3. (PDF) [file pone.0042086.s007.pdf]

**Table S7.** Microsatellites analyzed at 21q22.3.

| Microsatellite | Primers (5´→3´) | Amplicon (bp) | AT (ºC) | Repetition | Max. heterozy-gosity |
|---|---|---|---|---|---|
| **D21S1235** | S: CTTTCATGTGTGTCTACGGAT<br>AS: GGCTACTCTCTGCCCAGAT | 102-134 | 58 | Dinucleotides | 0.78 |
| **D21S1224** | S: GAAGGAGCTATACCCGGACT<br>AS: GCTAGTAGCTACCATATTGG | 140-154 | 55 | Dinucleotides | 0.83 |
| **D21S1411** | S: ATGATGAATGCATAGATGGAT<br>AS: AATGTGTGTCCTTCCAGGC | 266-314 | 58 | Tetranucleotides | 0.93 |
| **D21S1259** | S:GGGACTGTAATAAATATTCTGTT<br>AS: CACTGGCTCTCCTGACC | 202-224 | 53 | Dinucleotides | 0.67 |
| **D21S1912** | S: CCCTCATACAGATTTAAAACAC<br>AS: GAGCCCACCCTGGTAAC | 172-202 | 55 | Dinucleotides | 0.80 |
| **D21S171** | S: TAGGCCCTACTGCAATAATG<br>AS: CTTTATCTTCACACAGCTTC | 106-124 | 55 | Dinucleotides | 0.80 |
| **D21S1574** | S: GAAGACTGCTTGAGCACAGG<br>AS: TGCATTCCTGGTGTAAACCC | 168-182 | 62 | Dinucleotides | 0.78 |
| **D21S1446** | S: ATGTACGATACGTAATACTTGA<br>AS: GTCCCAAAGGACCTGCTC | 203-227 | 55 | Tetranucleotides | 0.78 |

*Microsatellites are ordered from the most centromeric to the most telomeric. Table shows primer sequences (S: Sense; AS: Antisense), amplicon length intervals, annealing temperatures (AT) used in the PCR, types of repetition, and maximum heterozygosities.*
