# Supplementary material for: Identification of Novel Deregulated RNA Metabolism-Related Genes in Non-Small Cell Lung Cancer
Source: PLoS One. 2012 Aug 2;7(8):e42086. doi: 10.1371/journal.pone.0042086 (PMC3410905; doi:10.1371/journal.pone.0042086)
Supplement: Table S8 — Demographic and clinical characteristics of the 213 patients selected from the cohort of adenocarcinoma patients (Shedden et al., 2008). (PDF) [file pone.0042086.s008.pdf]

Table S8. Demographic and clinical characteristics of the 213 patients selected from the cohort of adenocarcinoma patients (Shedden et al., 2008).

**Age - years**	
Median	65
SD	10
**Gender - n (%)**	
Female	116 (54%)
Male	97 (46%)
**Smoking status - n (%)**	
Nonsmoker	27 (13%)
Former	160 (78%)
Current	19 (9%)
NA	7
**Stage - n (%)**	
I	157 (74%)
II	35 (17%)
III	19 (9%)
IV	0 (0%)
NA	2

NA: not available
